# A 10 year retrospective study on oral salivary gland lesions in India

**DOI:** 10.6026/973206300211978

**Published:** 2025-07-31

**Authors:** Sivakumar R., Manju Mariam Stephen Mathunny, Padmakumar S.K

**Affiliations:** 1 Department of Oral pathology and Microbiology, Government Dental College, Thiruvananthapuram, India

**Keywords:** Salivary gland, lesions, tertiary care

## Abstract

703 oral salivary gland pathology cases from 2014 to 2023 out of 6,143 biopsies (11.4%) were analysed. Non-neoplastic lesions accounted for 89%, with mucocele being most common (84.2%). Among neoplasms, 59% were benign-primarily pleomorphic adenoma-and 41% were malignant, with mucoepidermoid carcinoma most frequent. The distribution patterns aligned with known literature, though malignant cases were comparatively higher.

## Background:

Salivary glands are the exocrine organs for saliva production and secretion that consist of three pairs of major salivary glands (parotid, submandibular and sublingual glands) as well as hundreds of minor salivary glands distributed in the lining mucosa of the upper aerodigestive tract [1]. Saliva is a highly complex mixture of water, organic, and non-organic materials. It aids in the lubrication of the oral cavity, mastication, swallowing, and protection of the mouth and teeth [2]. Salivary gland pathologies comprise an enormous spectrum of non-neoplastic (autoimmune, inflammatory, infections) and neoplastic disorders (benign and malignant) [3]. Salivary gland tumors, though relatively rare, have shown evolving trends in incidence, pathology, and management in recent decades. A review by Lin *et al.* highlighted that salivary gland cancers (SGCs) account for roughly 6% of all head and neck malignancies, with a gradual increase in incidence from 1.1 to 1.3 cases per 100,000 individuals in the U.S. between 1975 and 2015 [4]. Complementing these findings, Valstar *et al.* conducted a large-scale Dutch registry study on pleomorphic adenomas, the most common benign salivary gland tumors, reporting a standardized incidence of 4.2-4.9 per 100,000 person-years with a female predominance and an increasing annual trend among women [5]. It is estimated that the prevalence of inflammatory lesions of the salivary glands in the population is about 1.2%. [6]. Inflammatory changes associated with salivary gland stones are responsible for up to half of such pathologies [7]. Malignant tumors comprise 15 -25% of all parotid tumors, 37-43% of submandibular gland tumors and over 80% of minor salivary gland tumors [8]. Salivary gland diseases hold the interest of clinicians and pathologists due to their varied clinical presentation and histological diversity [3]. Due to their rarity, histological heterogeneity and classification changes of SGTs, epidemiological studies are difficult, but epidemiological data is important for diagnosis and management [9]. Epidemiologic studies have shown that the incidence and subgroup distributions of salivary gland neoplasms vary across the world, with diverse demographic results in different regions [10]. The biological behavior of salivary gland tumors, as noted by Ackerman and Del Regato, reflects their distinctive nature-where benign tumors often exhibit more aggressive tendencies than typical benign neoplasms, and malignant tumors tend to display a less aggressive course compared to most other malignancies [11]. Therefore, it is of interest to study on oral salivary gland lesions of a tertiary health care centre in India.

## Materials and Methods:

The study was conducted as retrospective chart review at the Department of Oral pathology and Microbiology, Government Dental College, Thiruvananthapuram. The reports of all salivary gland biopsies reported at the Oral pathology department from 2014 to 2023 were reviewed. The study was conducted for duration of 6 months. A structured clinical record proforma was used to gather information such as age, gender, site of lesion and histopathological diagnosis. The case records and reports were thoroughly analysed for clinicopathologic features like gender, age, site, clinical presentation and histopathology. Descriptive statistical analysis was performed using the computer software.

## Results:

A total of 6,143 biopsies reported to the department during the study period of which salivary gland biopsies were 703 which is about 11.4%. Out of the total 703 salivary gland biopsies, 627 cases were non-neoplastic lesions (89%) and 76 cases were neoplastic lesions (11%).Among neoplastic lesions, benign salivary gland tumours comprised of 45 cases (59%) and malignant tumours comprised of 31 cases (41%). Among non-neoplastic lesions, the most common was mucocele with 528 cases (84.2%). Sjogren's syndrome constituted 44 cases (7%) and Sialadenitis was about 32 cases (5.2%). Sialolithiasis constituted of 20 cases (3.2%). Necrotizing sialometaplasia and glandular hyperplasia constituted 1 case each (0.2%). Among benign salivary gland neoplasms, Pleomorphic Adenoma was the most common (n=36; 47.4%). Canalicular adenoma, Basal cell adenoma and Ductal papilloma were scored second with 2 cases each (2.6%). Cellular adenoma, Myoepithelioma and benign spindle cell tumour were 1 case each (1.3%).Among malignant salivary gland tumours, Mucoepidermoid carcinoma was the most prevalent with 18 cases (23.7%). Adenoid cystic carcinoma was the next with 6 cases (7.9%) (Table 1-3 - see PDF). Polymorphous adenocarcinoma constituted 4.0% and carcinoma ex-pleomorphic adenoma constituted 2.6%. Acinic cell carcinoma and Epithelial myoepithelial carcinoma constituted 1.3% each.Statistical analysis of our study revealed that, the mean age of non-neoplastic lesions was 26.7 (SD =16.5), benign lesions 42.4 (SD =15.9) and malignant lesions 44.6(SD =16.2) [p value: 0.027]. Non-neoplastic lesions were more common in males (52.56%) while the neoplastic lesions were more common in females (63%). Almost 95% of lesions occurring on palate were neoplastic on the other hand; about 99.6 % of lesions occurring on lower labial mucosa were non-neoplastic (Figure 1, 2 - see PDF).

## Discussion:

Among 6,143 biopsy cases reported, 11.4% were salivary gland pathologies. This was significantly higher when compared to other similar studies done by Sabarinath *et al.* [12] and Lawal *et al.* [13], which showed only 6%. The present study showed a predominance of non-neoplastic lesions (89%) while neoplastic lesions were comprised of 11%. Of these neoplastic cases, benign lesions were about 59% and malignant lesions were about 41%. In our study, the frequency of malignant salivary gland lesions was significantly higher when compared to other studies which showed around 20% malignant lesions [14, 15]. Of the non-neoplastic lesions, 84% were Mucocele. Out of which, mucous extravasation cyst was much higher (97.3%) when compared to mucous retention cyst (2.7%); this finding was consistent with the existing literature [16, 17]. Most of the reported cases of mucocele, had a history of trauma or habit of lip biting. Mucoceles were predominant among younger age groups, mostly 11 -20 years age group. Sex predilection was somewhat evenly poised with 55% and 45% from males & females respectively. Most common site of occurrence was lower labial mucosa (84.7%) followed by buccal mucosa (5.5%) and tongue (4.5%). These findings were in agreement with other similar studies [9, 18]. In case of sialadenitis, the study showed no age predilection and showed a male sex predilection of 59.3% while sialolithiasis was more commonly seen in elder age group (61-70 years) and more common in females. Almost 80% of sialolithiasis occurred in submandibular salivary gland which was consistent with existing literature [9, 19]. In our observation, Sjogren's syndrome was more common in people above 40 years ad showed strong female predilection of 80%.

It was also observed that primary Sjogren's syndrome was more common than secondary. The study showed that there was a significant increase in Sjogren's syndrome cases in the post-covid era. Thorough literature search has revealed that the monoclonal antibodies against the virus produced in recovered Covid 19 patients are cross reactive and capable of recognising nuclear antigens [20]. The results potentially implicate that SARS - COV -2 could be an environmental trigger for Sjogren's syndrome and responsible for its rise in the prevalence during the post- COVID time. As per our observation, the most common salivary gland neoplasm was Pleomorphic Adenoma with strong female predilection (70%) and commonly affecting middle age group of 41 - 50 years and the most common intraoral site of occurrence is palate (83.3%). Our findings were consistent with the existing literature [21, 22]. But most of the studies have shown that the parotid gland was the most common site of pleomorphic adenoma. In our study, pleomorphic adenomas affecting parotid gland were comparatively less, may be due such patients reporting to ENT or General Pathology departments. Other benign salivary gland lesions reported were adenoma, papilloma & myoepithelioma, but were comparatively less. In our study, the most common salivary gland malignancy was Mucoepidermoid carcinoma (MEC) which was consistent with the existing literature [22, 23]. MEC was more common among 31-40 years age group and showed almost equal gender predilection (1:1) with palate as the most common site of occurrence (72%). Adenoid cystic carcinoma ranked as next followed by Polymorphous adenocarcinoma and carcinoma ex-pleomorphic adenoma. Acinic cell carcinoma and epithelial myoepithelial carcinoma were comparatively rare.

## Conclusion:

Higher prevalence of salivary gland pathologies, both neoplastic and non-neoplastic, was noted. The prevalence of malignant salivary gland tumours was markedly higher in our population.

## Financial support and sponsorship:

Nil
